# Urinary Incontinence and Other Pelvic Floor Dysfunctions as Underestimated Problems in People under Forty Years: What Is Their Relationship with Sport?

**DOI:** 10.3390/life14010067

**Published:** 2023-12-30

**Authors:** Lorenza Bonaldi, Maria Vittoria Mascolini, Martina Todesco, Anna Zara, Camilla Rossato, Caterina Fede, Chiara Giulia Fontanella, Carla Stecco

**Affiliations:** 1 Department of Civil, Environmental and Architectural Engineering, University of Padova, Via F. Marzolo 9, 35131 Padova, Italy; martina.todesco.2@phd.unipd.it; 2 Department of Industrial Engineering, University of Padova, Via Venezia 1, 35121 Padova, Italy; mariavittoria.mascolini@phd.unipd.it (M.V.M.); chiaragiulia.fontanella@unipd.it (C.G.F.); 3 Department of Neuroscience, University of Padova, Via A. Gabelli 65, 35121 Padova, Italy; anna.zara.1@studenti.unipd.it (A.Z.); camilla.rossato.2@studenti.unipd.it (C.R.); caterina.fede@unipd.it (C.F.); carla.stecco@unipd.it (C.S.); 4 Centre for Mechanics of Biological Material (CMBM), University of Padova, Via F. Marzolo 9, 35131 Padova, Italy

**Keywords:** urinary incontinence, pelvic floor dysfunction, sport, gender, aging

## Abstract

Urinary incontinence is still an underestimated problem due to its anatomical complexity and social taboo. Most of the time, it is believed to affect predominantly the elderly female population, and the literature still lacks data on its presence in the younger and male populations. Its relationship with other pelvic floor dysfunctions (PFDs) and sport activity remains an open topic. Thus, the present study surveyed 342 subjects of both genders, ranging from 18 to 39 y/o and with different sport activity levels, to understand the prevalence of PFDs (such as haemorrhoids, anal fissures, involuntary urinary/faecal leakage, and urgency). The results also showed a significative prevalence in younger, sporty, and male people. Approximately one third of the population had urinary incontinence mostly during stress activities (sport activity: 17%, cough/sneeze: 13%). The statistical analysis confirmed a higher prevalence in the cases of a light (32%) and intense (41%) sport activity level and a protective role of sport if practiced between 5 and 10 h/week, with bodybuilding/CrossFit and running seeming to be the riskiest sports. The relationship with the other PFDs showed a statistically significant dependence with most of them, confirming that urinary incontinence cannot be considered a separate problem from the other PFDs.

## 1. Introduction

To date, urinary incontinence (UI) is estimated to have a worldwide prevalence from 1 to 39% in males and from 25 to 45% in females, with an increase across the adult life course and due to the global aging phenomenon in both sexes [1]. On the other hand, the worldwide prevalence of faecal incontinence is estimated to be 7.7% (range [2.0–20.7]%), with specific percentages in men and women of 8.1% (range [2.3–16.1]%) and 8.9% (range [2.0–20.7]%), respectively [2]. In addition, pelvic floor muscle disorders may lead to other complications, such as urinary urgency [2], faecal urgency, haemorrhoids, and anal fissures [3]. Regarding these other pelvic floor dysfunctions (PFDs), it is more difficult to estimate the impact since few patients seek medical care. A prevalence of frequent or urgent urination was shown in 24.3% patients in a cross-sectional study on older adults in China, with a higher percentage in the 70–84 y/o group (men: 33.3–34.8%, women 19.5–20.8%) [4]. Regarding faecal urgency, a survey using the medical records of constipated patients reported the rate of 70.8% episodes in the past 3 months [5]. According to the literature, haemorrhoids constitute a common anal pathology that affects 20–50% of the population [6]. In an international study performed on adults in 2020, the prevalence of haemorrhoids was 11%, with a slightly higher proportion of females (52% vs. 56%) and obese people (19% vs. 21%) and a higher mean age (44.9 vs. 46.8 y/o) in the cohort with the disease compared to the general population [7]. Other than this, in a retrospective analysis on USA patients treated for anal fissures (58% females, 42% males), 12% of the cases occurred in children ages 6–17 y/o, and the overall annual incidence, influenced by age and by sex, was shown to be significantly higher among females of 12–24 y/o and among males of 55–64 y/o [8].

However, in the literature, pelvic floor dysfunctions are mostly still considered in relation to a specific subset of the population, such as the elderly female population, and restricted to only specific aspects, such as UI [9,10,11,12]. The underestimated prevalence of overall PFDs proves that the impacted population do not consider them as significant pathologies and even that they do not express these discomforts out of shame, thus leading to a reduction in terms of prevention and treatments [1].

Concerning aetiology, in addition to pregnancy and delivery, there are many factors that could compromise the functionality of the pelvic floor in the global population. In these terms, sport has a valuable impact. In 2018, a systematic review on nulliparous adolescent and middle-aged women reported a prevalence of UI from 1 to 42.2% (median 20.1%) and with a higher value for women who exercised compared to those who did not [13]. Considering different sports, a review on female athletes showed an overall prevalence of UI as 25.9%, with the most prevalent high-impact sport being volleyball, with 75.6% [14]. Moreover, the impact of sports on UI in females (22.18 ± 6 y/o, BMI normal value: 24.60 ± 19.14 kg/m^2^) was also studied in 2021, and major symptoms were reported in cycling and running groups, while minor symptoms were reported for collective sports such as hockey [15]. In the same year, Rodriguez-Lopez et al. [16] investigated the same prevalence among different sport athletes, also including the difference between sexes (60.3% women, 39.7% men, mean age: 23.04 ± 7.16 y/o). Overall, 33% of the athletes (45.1% of the females, 14.7% of the males) had UI at a mean age of 23.75 ± 7.74 y/o, and the sports in which this symptom was most detected were rugby (80%), swimming (57.1%), hockey (43.5%), karate (45.5%), athletics (40.5%), orienteering (40%), and dancing (40%). In detail, 22.7% of the athletes reported they had experienced urine leakage while training (40.5% when jumping, 19.6% while running, and 20.2% in different situations) [16].

However, in general, there is a minimal amount of information concerning UI in male athletes, compared to the female population, as well as a lack, for both genders, of data linked to sport activity in relation to other pelvic floor dysfunctions. As proof of this concept, based on a review published in 2020 considering 100 studies on pelvic floor dysfunction among male and female athletes, only a paucity of authors investigated symptoms other than UI (two studies examined anal incontinence, one study explored pelvic organ prolapse, and two studies addressed pelvic pain), and only twelve investigated pelvic floor dysfunctions in male athletes [17].

For these reasons, the aim of the present work is to collect and analyze data on pelvic floor dysfunctions, focusing on urinary and faecal leakage/urgency, haemorrhoids, and anal fissures in a population under forty years of age, including both sexes. In addition, the impact of sport on these dysfunctional conditions is evaluated in terms of the sport activity (sedentary, volleyball/basketball, bodybuilding/CrossFit, running) and activity level (light, mild, intense, as clustered based on hours/week). Evidence of a significant incidence of PDFs in an apparently healthy cohort is key for both treatment and prevention management.

## 2. Materials and Methods

### 2.1. Survey Design

The survey investigated pelvic floor dysfunctions in both genders in relation to sport activity, and it was created based on a literature study focused on pelvic floor dysfunctions and working activities in the female population [18]. The essential aspects characterizing the pelvic floor in healthy conditions and the main variables of interest to investigate its dysfunctions were identified and, based on these, a new survey was formulated. To simplify data collection, the survey was divided into three main parts (Table 1): the first part pertained to the general information of the subjects (Part I: Subject description), the second part focused on the sport practiced by the subject (Part II: Sport), and the final part related to pelvic floor dysfunctions (Part III: Dysfunction). Concerning the third part, the question was whether each of the proposed dysfunctions had ever occurred; thus, the choice of answers was a dichotomic variable (Yes/No).

### 2.2. Participants

A specific link to the survey was created through the software Survio^®^ (www.survio.com) and was shared on social networks during 2022. The purpose of the study was clarified in the survey marking preview; in this way, the study population was voluntarily recruited. At prior, the eligibility criteria for inclusion in the study were the absence of co-morbidities or pharmaceutical treatments that could affect the investigated clinical situations (as clarified in the survey preview). All participants were asked to sign at prior a privacy form regarding the processing of personal data to be used in anonymous form for publication. The population inclusion criteria matched the following categories: sedentary, runner, volleyball or basketball player, bodybuilder, or CrossFitter. During post processing, some surveys were excluded from the study due to an age group criterion (inclusion range: 18–39 y/o), and/or due to consistency/lack of information in answers. Finally, the total sample size comprised 342 eligible candidates.

### 2.3. Statistical Analysis

The statistical analysis, including both descriptive and inferential statistics, was performed on the data collected from surveys using Minitab^®^ Statistical Software (Software, Minitab, State College, PA, USA 2010 Minitab, Inc., www.minitab.com). Data were described with mean ± standard deviation (SD). Meanwhile, the inferential statistic was based on the one-way analysis of variances (ANOVA) test and chi-squared test. As an example, considering the Sport activity level as the quantitative variable, the ANOVA test checked whether the mean was different among all other qualitative variables. For this test, the populations were assumed to be normally distributed according to the central limit theorem, while their equal variances were proven via Levene’s test. In addition, population samples were assumed as randomly and independently drawn. Meanwhile, the non-parametric chi-squared test was used to check the independence between the different qualitative variables. For all these tests, the confidence interval was assumed to be 95% (α = 0.05). In Table 2, a summary of the applied test methods is reported.

## 3. Results

### 3.1. Descriptive Statistics

The results of the descriptive statistical analysis are reported in Table 3, in terms of mean ± SD, ranges, and their frequency of occurrence. A categorization of the subjects was performed in terms of age by splitting them into two groups, young adult (243 subjects) and adult (99 subjects), with a threshold of 25 y/o. Based on the survey’s data, the body mass index (BMI) for each subject was computed, and then subjects were split into four ranges according to the categories determined by the World Health Organization [19] (underweight: 16–18 kg/m^2^, normal weight: 19–24 kg/m^2^, overweight: 25–29 kg/m^2^, and obese: 30–36 kg/m^2^). Regarding the activity levels (hours/week), the mean ± SD and the frequency for each range (light, mild, intense) are reported with all the sport activities considered together.

### 3.2. Inferential Statistics

#### 3.2.1. One-Way ANOVA Test

A one-way ANOVA test showed that, considering the Sport activity level as quantitative variable, the population means are different when the qualitative variable Urinary leakage during cough/sneeze is considered (Table 4). For all the other qualitative variables, no differences were found among the population means.

#### 3.2.2. Chi-Squared Test

Chi-squared tests were performed among all the qualitative variables of the survey, while the results are reported only for the statistically significant relationships obtained.

The results showed a statistically significant dependence (*p*-value < α) between the population age and the qualitative variable Sport activity, belonging to the second part of the survey (PART II), and Anal fissures and Haemorrhoids, belonging to the third part (PART III). In Table 5, the percentages of occurrence are fully reported for the considered ranges in the case of the Sport variables (the sum of percentages equals to 100%), while they are reported only for the positive (Yes) answer in the case of Dysfunction variables (in other words, we report only the percentage of the dysfunction that was detected).

Considering the variable Sport activity, chi-squared tests showed a statistically significant dependence (*p*-value < α) with Gender, Activity level, Urinary and Faecal leakage in general and During sport activity, as well as While coughing/sneezing (Table 6). In Table 6, the percentages of occurrence are fully reported for the considered ranges in the case of the Gender variable (the sum of percentages equals 100%), while they are reported only for the positive (Yes) answer in the case of the Dysfunction variables (in other words, we report only the percentage of the dysfunction that was detected).

A statistically significant dependence (*p*-value < α) with Gender and Urinary leakage in general was also found via chi-squared tests when the variable Sport activity level was considered. In addition to these variables, the hypothesis tests showed Haemorrhoids and Urinary leakage during sport activity as dependent on the Activity level (Table 7). In Table 7, the percentages of occurrence are fully reported for the considered ranges in the case of the Gender variable (the sum of percentages equals 100%), while they are reported only for the positive (Yes) answer in the case of the Dysfunction variables (in other words, we report only the percentage of the dysfunction that was detected).

Splitting the Light group into Extra light (less than 1 h/week; this does not mean fully Sedentary, but could be interpreted as a group of non-sporty people) and Light (1–4 h/week), to really distinguish sedentary people, and performing the chi-squared tests again, a statistically significant dependence (*p*-value < α) was found for Urinary leakage in general for the Yes answer, with the following percentages: 39% Extra light, 25% Light, 21% Mild, and 41% Intense.

The results from the chi-squared tests showed a dependence between Urinary leakage in general and the qualitative variables reported in Table 8. In Table 8, the percentages of occurrence are totally reported for the considered ranges in the case of the Gender variable (the sum of percentages equals 100%), while they are reported only for the positive (Yes) answer in the case of the Dysfunction variables (in other words, we reported only the percentage of the dysfunction that was detected).

The results of the chi-squared tests show a dependence between the population Gender and the qualitative variables reported in Table 9.

## 4. Discussion

The surveyed populations mostly included young people (24 ± 4.2 y/o), approximately equally distributed between males (46%) and females (54%) and with a BMI in the normal range (23 ± 2.8 kg/m^2^). Sedentary behavior was reported by 32% of respondents, while others claimed to practice a sport activity such as running (16%), volleyball/basketball (31%), or bodybuilding/CrossFit (21%), with an average sport activity level of 5.2 ± 4.5 h/week, as reported in Table 3. From the results of this investigation, a significative percentage of this cohort was found to suffer different pelvic floor dysfunctions (anal fissures, haemorrhoids, involuntary urinary or faecal leakage, and/or urgency) in accordance with their daily living activities. In general, one third of the population had claimed to have discomforts such as urgency. Specifically, faecal urgency was estimated to be 35%, although Singh et al. demonstrated a rate of 70.8% in constipated patients [5]. Meanwhile, urinary urgency was estimated to be 37%, higher than in the study of Zhang et al., which reported a value of 24.3% in Chinese populations, also considering the prevalence of frequent urination [4]. As regards urinary leakage, the results are in line with the ranges reported for the worldwide population by Toniolo et al. [1], as it was found to be 29% (during sport: 17%, cough/sneezing: 13%). For faecal leakage, the current study obtained a higher value (in general, 27% and during sport activity, 18%) than [1]; however, these authors did not mention any relationship with stress activity. See Table 3. In addition, an increment in dysfunctions was found with aging (e.g., anal fissures and haemorrhoids: 8.6% and 7.8% for subjects under 25; 16% and 24% for subjects over 25). See Table 5. For haemorrhoids, those outcomes are in line with the results of Sandler et al. [6], who reported a percentage in the range of 20–50%, and of Sheikh et al. [7], who also reported the prevalence in relation to gender, BMI, and aging (higher in females, obese people, and elderly people). The presence of dysfunctions in younger people was also confirmed by another study that showed that faecal leakage increases with age (5.7% for 15–34 y/o; 15.9% for >90 y/o) [1].

**Figure 1:**
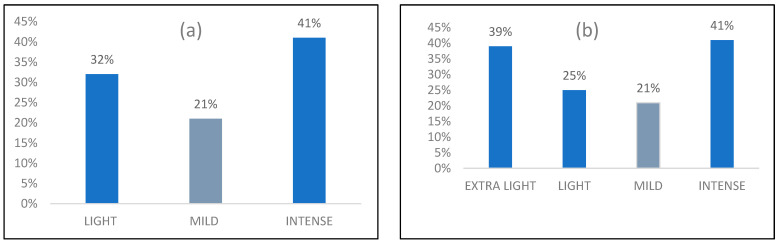
Sport activity level in relation to Urinary leakage (general) prevalence: (**a**) Light, Mild, and Intense, (**b**) with Extra-light group added.

Regarding sport activity, the present study shows a clear relationship with pelvic floor dysfunctions. In detail, one-way ANOVA tests showed that the presence of urinary leakage, such as while coughing/sneezing, is significantly associated with specific activity levels (hours/week). A lower sport activity level (3.8 ± 4.3 vs. 5.4 ± 4.4 h/week) was found in cases of subjects suffering such discomforts, triggering the idea of the protective role of sport if performed at a mild activity level (5–10 h/week), as reported in Table 4. Focusing on the younger adult population (18–25 y/o), the results obtained from statistical analysis proved that most of them are sedentary (35%). Meanwhile, the subjects over 25 y/o were revealed to be more sporty, with 32% taking part in volleyball/basketball and bodybuilding/CrossFit. Even if there is an increment in dysfunctions with aging, nonetheless, at least 7.8% of the younger people suffer from one pelvic floor dysfunction. See Table 5. Splitting the overall population into further subgroups in relation to the sport activity levels, the results show that practicing a mild sport activity is related to lower percentages of pelvic floor dysfunctions as urinary leakage, in general and during sport activity, and as haemorrhoids, compared to the Light and Intense level groups. Specifically, regarding the urinary leakage, its prevalence (for this study cohort) is reported in Figure 1. To investigate in more detail the Light activity level, we split it into two sub-groups (0–1 h/week and 1–4 h/week), confirming a higher prevalence of urinary leakage (in general conditions) for intense sport practitioners (41%) and non-sporty people (39%), and a protective role played by sport if practiced between 5 and 10 h/week. In any case, in relation to the risk associated with intense activities, this correlation should be better investigated to better understand the root causes. See Table 7.

From the quantity to the quality of each specific sport, running seem to be the riskiest sport for pelvic floor dysfunctions, in line with what was reported by Selecka et al. about running [15]. In the current study, runners showed a higher prevalence of faecal and urinary leakage during sport activity (32% and 28%, respectively), and in general (42% and 28%). See Table 6. Rodriguez-Lopez et al. [16] reported a lower urinary leakage in athletes during training such as running, at 19.6%. Regarding sedentary people, in the current study, these showed the highest percentage of urinary leakage in general (38% of them) and while coughing or sneezing (20%), compared to the sporty population, as reported in Table 6.

Looking at the relationship between urinary leakage (general) and other pelvic floor dysfunctions, the results of the chi-squared tests show a statistically significant dependence with most of them (urinary and faecal urgency, faecal leakage), thus confirming the anatomical and functional continuity between pelvic floor structures. Specifically, considering the population suffering from urinary leakage (general), 52% and 44% of them have urinary and faecal urgency, respectively. Moreover, 40% have faecal leakage in general, and 29% have it during sport activity. Furthermore, 50%, 35%, and 30% have urinary leakage, respectively, during sport activity, coughing or sneezing, and without activity. These outcomes confirm that UI cannot be considered a separate problem from other pelvic floor dysfunctions. See Table 8.

In addition, urinary leakage also showed a statistically significant dependence on gender, since 87% and 13% of the total population suffering from such a condition are, respectively, females and males, independent of age. See Table 8. Moreover, considering the overall female group, 30% and 23% of them suffer from urinary leakage during sport activity and coughing or sneezing, while, in the male group, 1.9% and 1.3% do. See Table 9. Also, De Mattos Lourenco et al. [20] reported UI as a common condition in female athletes. The higher prevalence of urinary leakage in females than in males was also demonstrated by Rogriguez-Lopez et al. for athletes [16]. The current study also shows that pelvic floor dysfunctions are an underestimated problem in the male population, since the mean prevalence within the group was estimated to be 7.2%, and the prevalence reached 17% for faecal leakage (in general). For females, the mean prevalence of pelvic floor dysfunctions was estimated to be 26%, higher than in the male population. See Table 9.

To conclude, this study contributes to estimating the prevalence of pelvic floor dysfunctions in declared healthy subjects (with an absence of comorbidities as an inclusion criterion), confirming that it is also an underestimated problem in under-40, sporty, and male subgroups. Urinary incontinence prevailed in approximately one third of the population, with higher percentages during stress activities (sport or everyday life situations such as coughing/sneezing). Among the considered sports, the riskiest have been revealed to be running and bodybuilding/CrossFit. However, sport has been demonstrated to have a protective role if practiced at a mild level in terms of hours/week. The results also confirm that urinary incontinence cannot be considered a separate problem from other PFDs.

These outcomes highlight the importance of investigating this topic to spread prevention (such as sport promotion and patient-specific training and education) and offer specific treatments, without underestimating these problems, and thus also reaching young and sporty populations of both sexes. Some limitations of the present study could be addressed, such as not having considered the correlation with comorbidities, pelvic pain, pregnancy or post partum, and sexual dysfunctions over time. Thus, further correlation with these aspects could be assessed as a future development.

## Figures and Tables

**Table 1:** Structure of the survey.

**Part I:** Subject description	
Age	
Gender	
Weight	
Height	
**Part II:** Sport	
**Activity** Running Bodybuilding/CrossFit Volleyball/basketball Sedentary	**Level** Light (0–4 h/week) Mild (5–10 h/week) Intense (11–27 h/week)
**Part III:** Dysfunction	
Anal fissures	Urinary leakage during sport activity
Haemorrhoids	Faecal leakage during sport activity
Urinary urgency (general)	Urinary leakage without activity
Urinary leakage (general)	Faecal leakage without activity
Faecal urgency (general)	Urinary leakage while coughing or sneezing
Faecal leakage (general)	Faecal leakage while coughing or sneezing

**Table 2:** Inferential statistic hypothesis test.

Hypothesis Test	H_0_	H_1_
	Accepted, If *p*-Value ≥ α	Accepted, If *p*-Value < α
One-way ANOVA	All population means are equal.	At least one population mean is different.
Chi-squared	The two variables are independent.	The two variables are dependent.

**Table 3:** Descriptive statistics for the overall survey variables.

Part	Variable	Mean ± SD	Range	Frequency (%)
Subject description	Gender	-	Male	46
			Female	54
	Age (y/o)	24 ± 4.2	18–25	71
			26–39	29
	BMI (kg/m^2^)	23 ± 2.8	16–18	8.0
			19–24	74
			25–29	17
			30–36	1.0
Sport	Sport activity	-	Sedentary	32
			Running	16
			Volleyball/basketball	31
			Bodybuilding/CrossFit	21
	Activity level (hours/week)	5.2 ± 4.5	Light (0–4 h)	51
			Mild (5–10 h)	38
			Intense (11–27 h)	11
Dysfunction	Haemorrhoids	-	-	13
	Anal fissures		-	11
	Urgency (general)		Urinary	37
	Urgency (general) Leakage (general)		Faecal	35
			Urinary	29
	Leakage (general)		Faecal	27
	Urinary leakage		During sport activity	17
			Without activity	9.4
			While coughing/sneezing	13
	Faecal leakage		During sport activity	18
			Without activity	8.1
			While coughing/sneezing	5.0

**Table 4:** Statistically significant differences calculated using a one-way ANOVA test for Sport activity level (hours/week).

Part	Variable	Occurrence	Activity Level (Hours/Week)
Dysfunction	Urinary leakage while coughing/sneezing	Yes	3.8 ± 4.3
		No	5.4 ± 4.4

**Table 5:** Percentages of qualitative variables found to be dependent on the population age.

Age				
Part	Variable	Range	18–25 y/o	>25 y/o
Sport	Sport activity	Sedentary	35%	25%
		Volleyball/basketball	31%	32%
		Bodybuilding/CrossFit	17%	32%
		Running	17%	11%
Dysfunction	Anal fissures	-	8.6%	16%
	Haemorrhoids	-	7.8%	24%

**Table 6:** Percentages of qualitative variables found to be dependent on the Sport activity.

Sport Activity						
Part	Variable	Range	Sedentary	Running	Bodybuilding/ CrossFit	Volley/ Basket
Subject description	Gender	Male	31%	30%	50%	65%
		Female	69%	70%	50%	35%
		Light	99%	7.5%	51%	23%
Sport	Activity level	Mild	1.0%	60%	39%	64%
		Intense	0.0%	32%	9.0%	12%
Dysfunction	Leakage (general)	Urinary	38%	28%	28%	20%
	Leakage (general) Urinary leakage	Faecal	28%	42%	27%	21%
		During sport activity	19%	28%	18%	9.3%
	Urinary leakage Faecal leakage	While coughing/sneezing	20%	5.7%	15%	8.4%
		During sport activity	13%	32%	20%	15%
	Faecal leakage	While coughing/sneezing	6.5%	1.9%	8.4%	0.0%

**Table 7:** Percentages of qualitative variables found to be dependent on the Sport activity level.

Sport Activity Level					
Part	Variable	Range	Light	Mild	Intense
Subject description	Gender	Male	40%	57%	32%
		Female	60%	43%	68%
Dysfunction	Haemorrhoids	-	18%	6.1%	11%
	Leakage (general)	Urinary leakage	32%	21%	41%
	Urinary leakage	during sport activity	16%	13%	38%

**Table 8:** Percentages of qualitative variables found to be dependent on the Urinary leakage (general), in the case of a positive (Yes) response.

Part	Descriptive Variable	Range	Urinary Leakage
Subject description	Gender	Male	13%
		Female	87%
Dysfunction	Urgency (general)	Urinary	52%
	Urgency (general)	Faecal	44%
	Leakage (general)	Faecal	40%
	Urinary leakage	During sport activity	50%
	Urinary leakage	While coughing/sneezing	35%
	Urinary leakage	Without activity	30%
	Faecal leakage	During sport activity	29%

**Table 9:** Percentages of qualitative variables found to be dependent on Gender.

Gender				
Part	Descriptive Variable	Range	Female	Male
Dysfunction	Anal fissures	-	15%	6.4%
	Leakage (general)	Faecal	35%	17%
	Urinary leakage	During sport activity	30%	1.9%
	Urinary leakage	While coughing/sneezing	23%	1.3%
	Faecal leakage	During sport activity	25%	9.6%
Mean	Total		26%	7.2%

## Data Availability

The data are not publicly available due to restrictions.
